# Are open-identity donors prepared for release of their identity? Long-term follow-up of a national sample of oocyte and sperm donors

**DOI:** 10.1093/humrep/deaf134

**Published:** 2025-07-31

**Authors:** Claudia Lampic, Emilia Thorup, Marie Bladh, Elizabeth Nedstrand, Xana Brinck, Agneta Skoog Svanberg, Gunilla Sydsjö

**Affiliations:** Department of Psychology, Umeå University, Umeå, Sweden; Department of Psychology, Lund University, Lund, Sweden; Department of Obstetrics and Gynaecology in Linköping and Division of Children’s and Women’s Health, Department of Biomedical and Clinical Sciences, Faculty of Medicine and Health Sciences, Linköping University, Linköping, Sweden; Department of Obstetrics and Gynaecology in Linköping and Division of Children’s and Women’s Health, Department of Biomedical and Clinical Sciences, Faculty of Medicine and Health Sciences, Linköping University, Linköping, Sweden; Department of Psychology, Umeå University, Umeå, Sweden; Department of Women’s and Children’s Health, Uppsala University, Uppsala, Sweden; Department of Obstetrics and Gynaecology in Linköping and Division of Children’s and Women’s Health, Department of Biomedical and Clinical Sciences, Faculty of Medicine and Health Sciences, Linköping University, Linköping, Sweden

**Keywords:** donor conception, identity-release, donor preferences, genetic link, post-donation contact, support needs

## Abstract

**STUDY QUESTION:**

What are the perspectives of oocyte and sperm donors 14–17 years post-donation on the release of their identity and potential contact with donor-conceived offspring (DCO)?

**SUMMARY ANSWER:**

Most oocyte and sperm donors wanted to be notified about future releases of their identity and were positive towards contact with DCO, but more than half expressed a need for support in relation to potential contact.

**WHAT IS KNOWN ALREADY:**

Worldwide, an increasing number of individuals conceived by open-identity donation are reaching an age where they may request donor identity. Little is known regarding donors’ preparedness for identity-release and potential contact with DCO.

**STUDY DESIGN, SIZE, DURATION:**

This study is part of the ‘Swedish Study on Gamete Donation’ (SSGD), a longitudinal multicentre study including oocyte and sperm donors at all clinics performing gamete donation in Sweden. Consecutive recruitment during a 3-year period (2005–2008) resulted in an initial sample of 299 donors (80% response rate). The present study concerns the fifth wave of data collection of the SSGD conducted 14–17 years post-donation, with very high response rates (oocyte donor 83%, sperm donor 92%). Following exclusion of donors who knew the recipients and/or knew that the donation had not resulted in a living child, the final sample comprised 100 oocyte donor and 91 sperm donor.

**PARTICIPANTS/MATERIALS, SETTING, METHODS:**

Fourteen to seventeen years after having participated in open-identity donation of their gametes, participants completed a postal survey with study-specific questions. Questions concerned preferences related to the release of their identity to DCO, attitudes towards future contact with people conceived from their donations, need for support regarding potential contact, attitudes towards the perceived importance of the genetic link between parent and child, and openness about having donated oocytes or sperm. Chi-square tests, independent *t*-tests, and Mann–Whitney *U*-tests were used to compare responses between oocyte and sperm donors. Multinomial logistic regression was used to identify factors associated with donors’ attitudes towards future contact with DCO and need for support. Content analysis was used to analyse free-text responses.

**MAIN RESULTS AND THE ROLE OF CHANCE:**

Almost all oocyte and sperm donors wanted to be notified about requests for their identity (93%). A majority had positive (71%) or neutral (19%) attitudes towards contact with DCO, but a small group was negative (10%), and more than half wanted support related to potential contact (59%). Free-text responses indicated that donors took the interests of both the DCO and their own family members into account when considering future contact. Donors’ attitudes towards contact with people conceived from their donations and donors’ need for support were not predicted by socio-demographic factors such as donors’ gender and legal children, nor by the perceived importance of the genetic parent–child link. While oocyte and sperm donors displayed similar perspectives on most outcomes, oocyte donors were found to be more open about having donated to all people except partners (All *P*-values <0.05) and sperm donors placed a higher value on the genetic parent–child link (*P* = 0.005).

**LIMITATIONS, REASONS FOR CAUTION:**

While the multicentre design and high response rates strengthen the external validity of our findings, the results may not be generalizable to originally anonymous donors. The scope of the qualitative analysis was limited due to the restricted number of free-text responses. An interview format may be needed to further explore donors’ thoughts and feelings regarding potential contact with DCO.

**WIDER IMPLICATIONS OF THE FINDINGS:**

At a time when increasing numbers of donor-conceived people can request donor-identifying information, our finding that donors generally are positive or neutral towards being contacted by DCO is encouraging. Long-term support of open-identity donors should include notification about requests for their identity and access to counselling and information about handling potential future contact with people conceived from their donations.

**STUDY FUNDING/COMPETING INTEREST(S):**

The study has received financial support from the Swedish Research Council (grant number 2021-03174), the Swedish Research Council for Health, Working Life and Welfare, and grants from the Swedish state under an agreement between the Swedish Government and the County Councils, ALF Grants, Region Östergötland. The authors have no conflicts of interest to declare.

**TRIAL REGISTRATION NUMBER:**

N/A.

## Introduction

During the past decades, open-identity gamete donation has become increasingly common and in several countries donor-conceived persons (DCPs) are now reaching the age when they can request donor-identifying information (Blyth and Frith, 2015; Indekeu *et al.*, 2021). While all open-identity donors have accepted that their identity will be released to DCPs on request in the future, there is limited knowledge about their level of preparedness for disclosure of their identity and potential contact attempts by donor-conceived offspring (DCO) (Skoog Svanberg *et al.*, 2020).

Although open-identity donation has been available for some time, few studies have reported on the number of persons who obtain donor-identifying information. In two longitudinal studies from the USA, a third of the young adults had requested sperm donors’ identity (Scheib *et al.*, 2017) and had contacted them (Koh *et al.*, 2020). In Sweden, only 7% of those eligible had requested information about open-identity sperm donors (Lampic *et al.*, 2022) and few had made any contact attempts. While men and women donating in open-identity programs are under no obligation to engage in such contact, research shows that most are open to being contacted by DCO (Daniels *et al.*, 2005; Ekerhovd *et al.*, 2008; Isaksson *et al.*, 2014; Lampic *et al.*, 2014; Graham *et al.*, 2016; Scheib *et al.*, 2017; Miettinen *et al.*, 2019). Both oocyte and sperm donors report predominantly positive or neutral attitudes towards contact, but a few do not want to be contacted. Negative or apprehensive attitudes towards contact with DCO can be related to concerns about potential negative consequences for the DCP’s family (Miettinen *et al.*, 2019) or for the donor’s own family (Daniels *et al.*, 2012; Kirkman *et al.*, 2014). Both oocyte and sperm donors have been reported to desire support regarding potential contact with people conceived from their donations (Isaksson *et al.*, 2014; Visser *et al.*, 2016), which may involve advice about informing and involving members of their own family. Previous results from large surveys indicate that most sperm donors discuss the donation with their partner (Daniels *et al.*, 2012; Pennings *et al.*, 2021) and that most oocyte and sperm donors plan to inform their legal children about having donated (Daniels *et al.*, 2012; Miettinen *et al.*, 2019).

How donors view the act of donating oocytes or sperm seems to vary quite substantially. Some regard themselves strictly as ‘providers of a cell’ whose job is finished after the donation (Kirkman *et al.*, 2014; Graham *et al.*, 2016; Miettinen *et al.*, 2019). Others refer to DCO as ‘their children’, report feelings of responsibility, or show an interest in potential physical and behavioural similarities (Jadva *et al.*, 2010b; Daniels *et al.*, 2012; Kirkman *et al.*, 2014; Visser *et al.*, 2016). Men in general have been found to place a greater value on the genetic relationship between parent and child compared to women (Skoog Svanberg *et al.*, 2003; Isaksson *et al.*, 2011) but it is unknown if this also applies to gamete donors. As part of a larger sociological study on the medical market of selling gametes in the USA, interviews with 39 oocyte and sperm donors showed that more men than women considered themselves as parents to potential offspring from their donations (Almeling, 2011). These findings were discussed in relation to differences in the practices of the included egg and sperm banks. Most of the men had no information about the recipients of their donations, but they were all identifiable to any future offspring. In contrast, the women had donated anonymously but many had been in contact with the recipients, and their focus seemed to be on ‘giving a gift’ to the intended mothers (Almeling, 2011). It is unknown whether donors’ perceived importance of the genetic bond between parent and child is related to their attitudes towards contact with DCO.

As more and more DCPs are approaching the age of eligibility for donor-identifying information, the number of such requests is expected to rise. In addition, in several legislations previously anonymous donors voluntarily agree to make their identity available to DCO (Daniels *et al.*, 2012; Blyth *et al.*, 2017; Indekeu *et al.*, 2022). Oocyte and sperm donors make important contributions with life-long implications and it is therefore important to investigate their perspectives, needs, and preferences related to the release of their identity to DCPs. For example, it has been recommended that clinics contact donors in advance of the release of their identity (Scheib *et al.*, 2017; Lampic *et al.*, 2022; Widbom *et al.*, 2024), as this would give the donor time to reflect about potential future contact, and to inform family members. However, being notified about DCO requesting one’s identity may also have negative consequences, such as the risk of disappointment in case DCPs choose not to make contact (Isaksson *et al.*, 2014).

The present study was conducted in Sweden, where legislation mandating open-identity gamete donation was enacted in 1985. DCPs are entitled to obtain donor-identifying information at ‘mature age’, which is typically interpreted as 18 years of age (Stoll, 2008). Only altruistic donation is allowed but donors are reimbursed with a fixed sum intended to compensate for the costs associated with donating. All prospective donors undergo a mandatory psychosocial evaluation following national guidelines, conducted by a psychosocially trained counsellor. Donors have access to additional counselling services before and during the donation. Each clinic is free to determine its own counselling policy. Some changes in routines have occurred since the participants in the current study conducted their donations. For example, contemporary donors are informed that direct-to-consumer DNA-tests may entail earlier revelation of their identity or that family members find out about the donation (Stenfelt *et al.*, 2023). There is no national donation register in Sweden and matters regarding identity-release and information requests from donors and DCPs are handled by the clinics. Clinics do not routinely inform donors about the birth of children from their donations, but donors may contact clinics to enquire about this. While a recent study of identity-release in Sweden indicates that some clinics, but not all, notified donors in connection with requests for their identity (Lampic *et al.*, 2022), newly issued practice guidelines recommend clinics to notify donors of such requests and to provide post-donation counselling (Stenfelt *et al.*, 2023). As in many other countries, the subject of donor conception has received a lot of media coverage and public debate in Sweden in the past few years.

The present study concerns a follow-up of open-identity oocyte and sperm donors 14–17 years after donating and focuses on donors’ perspectives related to the future release of their identity to DCO. The study’s aims were:

1. To investigate donors’ preferences and attitudes regarding the release of their identity and future contact with DCO, and to compare oocyte and sperm donors in this regard.
2. To compare oocyte and sperm donors’ perceived importance of genetic parent–child link and openness about their donations.
3. To identify factors associated with donors’ attitudes towards future contact with DCO and need for support.

## Materials and methods

### Study population

The Swedish Study on Gamete Donation (SSGD) is a longitudinal multicentre study that includes women and men who were accepted as either donors or recipients of gametes at any of the seven Swedish clinics performing gamete donation in 2005–2008. This entails that all individuals donating gametes within the Swedish health care system at that time were approached regarding participation. Details of the study design and methods for inclusion and data collection for the gamete donors have previously been described (Sydsjö *et al.*, 2011, 2012, 2023; Skoog Svanberg *et al.*, 2012, 2013; Isaksson *et al.*, 2014; Lampic *et al.*, 2014). In short, donors have completed postal questionnaires at five waves of data collection: following acceptance (T1), 6 months post-donation (T2), 1 year post-donation (T3), 4–8 years post-donation (T4), and 14–17 years post-donation (T5). The fifth wave of data collection for gamete donors was performed in 2021–2022. The donors were sent a letter including study information, a consent form, and a questionnaire. Non-responders were sent up to two reminders; no incentives were provided. The SSGD project was approved by the Regional Ethical Review Board in Linköping and all participants provided informed consent.

Of all donors initially approached regarding participation in the SSGD, 83% of oocyte donors (181/217) and 76% of sperm donors (118/156) accepted and completed data assessment at T1. For the fifth wave of data collection, some of the initial participants were excluded due to active drop-out at previous data collection occasions (n = 8), being deceased (n = 3) or no available postal address in the Swedish Population Register (n = 15). Of the 273 donors approached for the current follow-up, 83% of oocyte donors (141/169) participated, as did 92% of sperm donors (96/104). As the main aim of the present study was to investigate donors’ attitudes related to the release of their identity to DCO, the following groups were excluded from analyses: participants who reported that they know the recipient couple, i.e. had participated in known/directed donation (18 oocyte and 4 sperm donor), and donors who stated knowing that their donation had not resulted in a living child (23 oocyte and 1 sperm donor). Thus, the present study included 100 oocyte donors and 91 sperm donors.

Attrition analyses were performed with respect to socio-demographic variables assessed at acceptance (T1) and donation-related issues 6 months post-donation (T2), as previously reported (Sydsjö *et al.*, 2023). In short, drop-outs did not differ from those who participated at long-term follow-up (T5) with respect to socio-demographic variables (except more often having biological children) or their perceptions of the genetic parent–child link and the possibility of being contacted by DCO.

### Data collection and measurements

The questionnaire at the fifth wave of data collection included socio-demographic variables (e.g. educational level, occupation, family situation), study-specific items concerning perceptions of donation-specific issues (e.g. information about the outcome of one’s donation), and the Hospital Anxiety and Depression Scale (HADS). Results of donors’ perceptions of the donation, financial compensation, and psychological distress (HADS) have been reported previously (Sydsjö *et al.*, 2023).

#### Preferences related to clinic’s identity-release to DCO

Participants’ preferences related to identity-release to DCO were measured with two items. The first item, which has previously been used at the fourth wave of the SSGD (Isaksson *et al.*, 2014), concerned if they want to be informed by the clinic when a DCP requests information about them. There were three response alternatives (‘No, I do not want to be informed’; ‘Yes, I want to be informed before my identity is released’; ‘Yes, I want to be informed after my identity has been released’). The second item concerned if participants want the clinic to inquire about their attitude towards contact with DCO (in order to convey this information to DCPs). Response alternatives were: ‘No, I do not want the clinic to inquire about that’; ‘Yes, I would like to state that I prefer not to be contacted’; ‘Yes, I would like to state that I am open to contact by DCO’; ‘I am unsure’.

#### Attitudes towards potential future contact with DCO

Participants’ attitudes regarding future contact with DCO were assessed with two items previously used at the second, third (Lampic *et al.*, 2014), and fourth wave of the SSGD (Isaksson *et al.*, 2014). The items concern attitudes towards being contacted by a DCO and towards the DCP meeting the donor’s family. Participants were asked to indicate their responses on a 5-point Likert scale from ‘totally agree’ to ‘totally disagree’, which were categorized into ‘Agree’, ‘Neutral’, and ‘Disagree’. In addition, participants were given the opportunity to elaborate on their answers in an open-response format.

#### Need for support regarding potential future contact with DCO

Donors’ need for support was investigated by an item previously used at the fourth wave of the SSGD (Isaksson *et al.*, 2014). Donors were asked to indicate whether they would like advice regarding future contact with DCO. Participants were provided with four response alternatives (‘No’; ‘Yes, written information’; ‘Yes, personal counselling’; ‘Don’t know’) and could also elaborate on their answer in an open-response format.

#### Importance of the genetic link between parent and child

Attitudes towards the perceived importance of the genetic link between parent and child were assessed by four items, two of which had previously been used by this research group (Skoog Svanberg *et al.*, 2003; Isaksson *et al.*, 2011). The items were: ‘The genetic bond between father and child is important’, ‘The genetic bond between mother and child is important’, ‘The love for a child does not have anything to do with genes’, and ‘Parents are those who live with and take care of a child’. Recipients were requested to indicate their responses on a five-point Likert scale from ‘Agree totally’ to ‘Disagree totally’. After reversing the scoring of the first two items, scores were summarized into an index variable, allowing scores to range between 0 and 16, with higher values indicating more importance being given to the genetic parent–child link. Cronbach’s Alpha for the index variable was 0.82 for sperm donors and 0.73 for oocyte donors.

#### Openness about having donated oocytes/sperm

Donors’ level of openness about their donation was assessed by a modified instrument previously used with donor gamete recipients (Isaksson *et al.*, 2011). Participants were presented with a list of potential confidants (e.g. ‘partner’, ‘children’, ‘friends’) and asked to indicate which individuals they had told about their donation. For the variables partner and child, only responses from donors who reported having a partner/child were included in the analysis.

### Data analysis

Pearson’s chi-square statistic or Fisher’s exact test was used to analyse categorical variables. Groupwise comparisons were conducted by independent samples *t*-tests or Mann–Whitney *U*-tests, depending on distribution and level of measurement. Two multinomial logistic regression analyses were conducted, with positive attitude towards future contact (‘Agree’, ‘Neutral’, ‘Disagree’) and need for support (‘Yes’, ‘No/Unsure’) as outcome variables and donation type (oocyte donation, sperm donation), education (University, Other), existence of legal children (‘Yes’, ‘No’), knowledge of donation outcome (‘Yes’, ‘No’), and scores on the genetic importance index as predictor variables. All analyses were performed using SPSS version 27, (SPSS IBM Inc., Armonk, NY, USA). Statistical significance was defined as *P* < 0.05 (two-sided). Content analysis as described by Erlingsson and Brysiewicz (2017) was used to analyse free-text responses regarding donor attitudes towards contact with DCO. Meaning units were identified and labelled with codes and grouped into categories and subcategories. To allow for identification of potential distinctive features of each donor group, initial analysis was performed separately for responses from sperm and oocyte donors. As the initial categories and subcategories created based on oocyte and sperm donors’ comments were highly similar, the decision was made to merge the material when finalizing the analysis.

## Results

### Participant characteristics

Sperm and oocyte donors were on average 49 and 45 years old at T5 (Table 1). A majority had completed university education, and this was more common among sperm than oocyte donors (*P* = 0.010). About a third reported living with the same partner as at the time of donation, with the remainder either living with a new partner or being single. A majority of donors had legal children, with ages ranging between 0 and 43 years. In comparison to sperm donors, a higher percentage of oocyte donors had children (*P* = 0.004) and were parents of adult children (*P* = 0.027). About half of the donors knew that their donations had resulted in the birth of children, whereas the other half was unaware of the donation outcome.

**Table 1.** Participant characteristics.

	Sperm donors N = 91	Oocyte donors N = 100	
	n (%)	n (%)	*P*-value
Age at follow-up,			
Median (min–max)	47 (34–72)	46 (35–52)	<0.001^1^
Mean/SD	48.9/7.8	44.9/4.6	<0.001^4^
Same partner as when donating			0.086^2^
Yes	24 (26.4)	40 (40.0)	
No, new partner	28 (30.8)	33 (33.0)	
No, I’m single	26 (28.6)	20 (20.0)	
Other^5^	13 (14.3)	7 (7.0)	
Educational level			0.010^3^
Secondary	14 (15.4)	31 (31.7)	
Tertiary	77 (84.6)	67 (68.4)	
Children			0.004^2^
No	29 (33.0)	15 (15.0)	
Yes^6^	59 (67.0)	85 (85.0)	
Age of children, at time of follow-up			0.027^2^
All children <18 years old	30 (52.6)	29 (34.5)	
All children ≥18 years old	7 (12.3)	25 (29.8)	
Children both age groups	20 (35.1)	30 (35.7)	
Awareness of donation outcome^7^			0.040^2^
Yes	49 (53.8)	39 (39)	
No	42 (46.2)	61 (61)	

1 Mann–Whitney *U*-test.

2 Pearson’s chi-square test.

3 Fisher’s exact test.

4 *t*-test.

5 Other includes one widower, one newly separated, and the others are those who were single at time of donation.

6 Of which three donors only have adoptive or stepchildren (two oocyte donors and one sperm donor) and 118 donors only have biological children (71 oocyte donors and 47 sperm donors).

7 I.e. knows whether the donation resulted in the birth of a child/-ren.

### Preferences related to identity-release, attitudes towards future contact, and need for support

The vast majority of donors stated that they would like to be informed by the clinic when a DCP requests information about them. Of those who did, fairly equal numbers preferred to be notified prior to versus after the release of their identity (Table 2). A majority also indicated that they would like the clinic to convey to the DCP that they were open to contact, whereas around 6% wanted the clinic to convey that they were *not* open to contact. Most donors reported a positive attitude towards being contacted by DCO, as well as to DCO meeting their family. About 60% of all donors indicated a need for written information/advice or counselling regarding future contact with DCO. Free-text comments provided to this item conveyed a need for advice regarding communication with DCPs, while others would like information about the legal framework and potential rights/obligations. Oocyte and sperm donors did not differ significantly in terms of preferences for identity-release, attitudes towards, or need for support regarding future contact with DCO.

**Table 2.** Preferences related to identity-release, attitudes towards future contact, and need for support.

	Sperm donors N = 91	Oocyte donors N = 100	
	n (%)	n (%)	*P*-value
Would you like the clinic to inform you when a (adult) child from your donation requests identifying information about you			0.220^1^
No, I do not want to be informed	9 (10.5)	4 (4.2)	
Yes, I want to be informed before my identity is released	38 (44.2)	50 (52.6)	
Yes, I want to be informed after my identity has been released	39 (45.3)	41 (43.2)	
Would you like the clinic to inquire about your attitude towards contact with donor-conceived offspring?			0.230^2^
No, I do not want the clinic to inquire about that	5 (5.9)	6 (6.2)	
Yes, I would like to state that I prefer not to be contacted	4 (4.7)	5 (5.2)	
Yes, I would like to state that I am open to contact	64 (75)	81 (84)	
I am unsure	12 (14)	5 (5.2)	
I think it is positive that I might be approached by a child after 18 years			0.858^2^
Agree	61 (70.9)	69 (71.1)	
Neutral	17 (19.8)	17 (17.5)	
Disagree	8 (9.3)	11 (11.3)	
I am positive towards a child meeting my family (e.g. my children) if he/she wishes			0.432^2^
Agree	51 (60.7)	66 (68.0)	
Neutral	16 (19.0)	12 (12.4)	
Disagree	17 (20.2)	19 (19.6)	
Would you like advice regarding future contact with children from your donation?			0.309^2^
No	27 (31.4)	23 (24.0)	
Yes, written information	22 (25.6)	35 (36.5)	
Yes, personal counselling	27 (31.4)	24 (25.0)	
Don’t know	10 (11.6)	14 (14.6)	

1 Fisher’s exact test.

2 Pearson’s chi-square test.

Due to partially missing values the totals do not sum up to population numbers. Also, due to rounding percentages may not sum up to 100%.

Separate multiple logistic regressions were conducted to identify predictors of a positive attitude towards contact with DCO and of the need for support. The results showed no significant associations between donation type, level of education, existence of legal children, knowledge of donation outcome, or scores on the genetic importance index and the outcomes (Table 3).

**Table 3.** Multiple logistic regression on attitude towards future contact and need for support, each outcome modelled separately, with odds ratios (ORs) and corresponding 95% CIs.

	Attitude towards future contact		Need for support
	Positive vs Neutral OR (95 % CI)	Positive vs Negative OR (95 % CI)	Yes^1^ vs No/unsure^2^ OR (95 % CI)
Donation type			
Oocyte	*Reference*	*Reference*	*Reference*
Sperm	1.74 (0.74–4.06)	1.54 (0.50–4.72)	0.71 (0.36–1.38)
Education			
Elementary/High school	2.34 (0.92–5.94)	3.03 (0.98–9.39)	0.80 (0.38–1.70)
College/University	*Reference*	*Reference*	*Reference*
Legal Children			
No	1.14 (0.44–2.91)	0.19 (0.02–1.58)	1.76 (0.79–3.92)
Yes	*Reference*	*Reference*	*Reference*
Aware of donation outcome^3^			
No	2.22 (0.92–4.85)	2.79 (0.89–8.73)	0.68 (0.36–1.28)
Yes	*Reference*	*Reference*	*Reference*
Genetic Importance	0.89 (0.78–1.02)	0.84 (0.70–1.01)	0.98 (0.89–1.08)

1 Includes responses ‘Yes, written information’ and ‘Yes, personal counselling’.

2 Includes responses ‘No’ and ‘Don’t know’.

3 I.e. knows whether the donation resulted in the birth of a child/-ren.

#### Qualitative results of donor attitudes towards future contact

About half of donors (56%) provided free-text responses regarding their views on potential future contact with DCO. Content analysis yielded three main categories: ‘Personal contact desires’, ‘Prioritizing needs of the DCP’, and ‘Managing family involvement’ (Table 4). The category ‘Personal contact desires’ concerned different aspects of donors’ own interest in future contact and included three subcategories. The first, ‘Interested’, comprised quotes expressing curiosity and excitement in the prospect of meeting DCO. The subcategory ‘Open to it’ comprised expressions of a more passive openness to contact, reflecting an attitude of ‘if it happens, it happens’ without stating any clear preferences. Finally, the quotes in the subcategory ‘Disinterested’ clearly expressed a lack of desire or interest in any contact with DCO. The category ‘Prioritizing needs of the DCP’ comprised quotes that expressed an overarching attitude of emphasizing the DCO’s preferences regarding contact. Some noted that, as DCPs had not chosen their mode of conception, their needs and wishes were considered most important. Others addressed DCPs’ potential interest in their genetic heritage and stated a willingness to supply such information. The final category, ‘Managing family involvement’, concerned how the needs and wishes of the donor’s own family members were considered important with regards to prospective contact with DCPs. Some conveyed a sense of protection of their families by stating a wish to meet DCO alone before deciding on potential contact with family members. Others expressed a wish to respect family members’ preferences that may differ from their own.

**Table 4.** Oocyte donors’ and sperm donors’ thoughts and feelings regarding future contact with donor-conceived offspring presented as categories and subcategories with illustrative quotes.

Category	Subcategory	Meaning units
Personal contact desires	Interest	‘I hope that the child will contact me. I am curious’ (oocyte donor)
		‘If no donor child ever contacts me I would probably be disappointed’ (oocyte donor)
		‘I would find it really interesting to meet the children my donation resulted in’ (sperm donor)
	Open to it	‘All children are welcome’ (oocyte donor)
		‘I don’t consider it a problem that I might be contacted’ (sperm donor)
		‘I don’t mind meeting the child’ (sperm donor)
	Disinterested	‘I have no interest in meeting children from my donations’ (oocyte donor)
		‘I have no need whatsoever to meet or establish a relationship with children who actually are not ‘mine’’ (sperm donor)
		‘I don’t have any need or longing to meet the child’ (oocyte donor)
Prioritizing needs of the donor-conceived person		‘I hope they are happy living their lives, and that they don’t care about me. But if they want to, they may contact me’ (sperm donor)
		‘The children’s rights are most important’ (oocyte donor)
		‘If a child feels a need to meet their biological father there might be some value to a meeting’. (sperm donor)
Managing family involvement		‘[I am] positive towards [donor-conceived offspring] meeting my family, but not the first time’ (oocyte donor)
		‘It depends a lot on how my family views it’ (oocyte donor)
		‘I am positive [to contact] but my wife may be of another opinion’ (sperm donor)

### Importance of genetic link between parent and child

**Figure 1.**
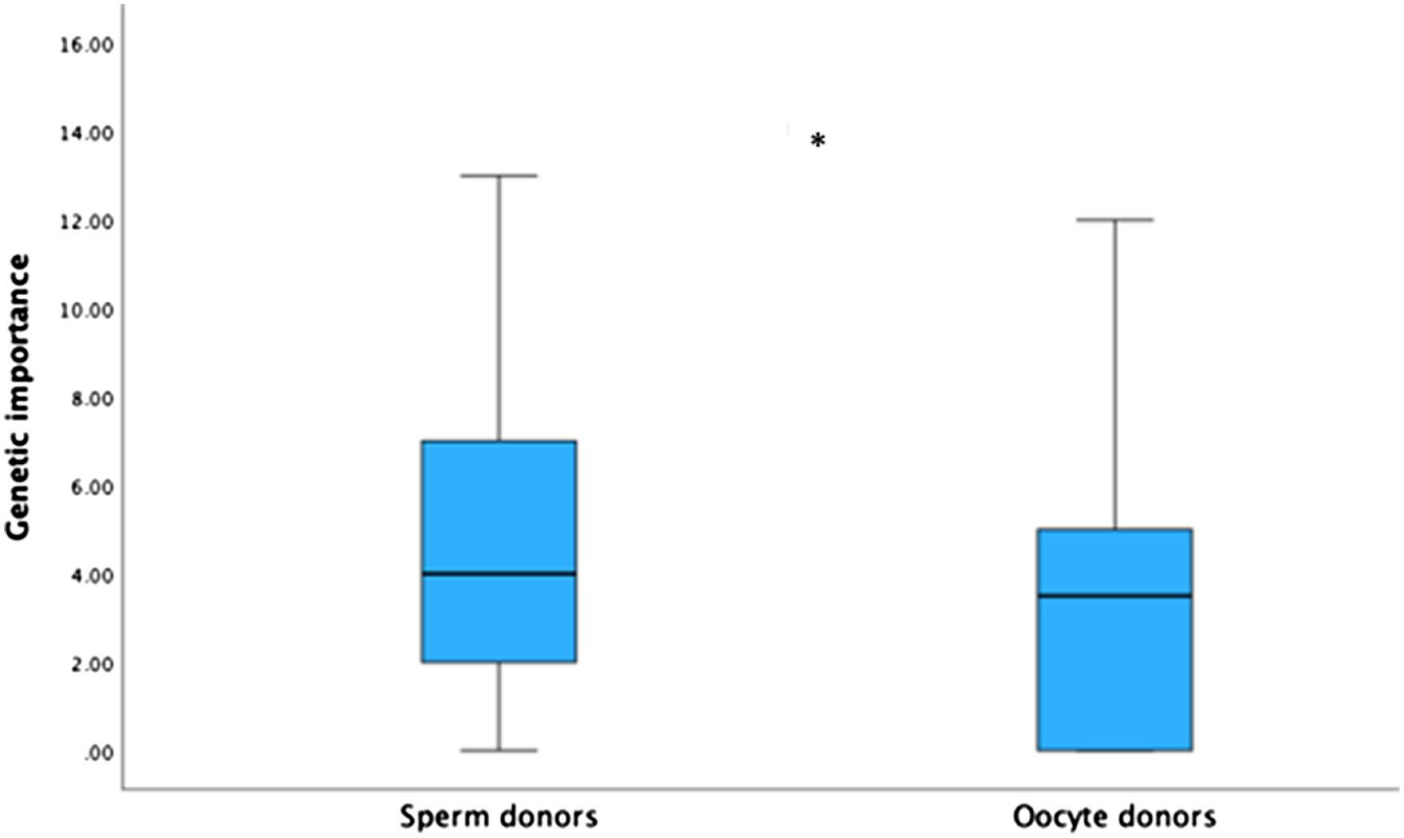
**Sperm and oocyte donors’ scores on the genetic importance index.** Scores could vary between 0 and 16. Box plots show median, the 75th and 25th quartiles, and the whole data range. *denotes statistical significance at the 0.005 level.

Sperm donors rated the importance of the genetic parent–child link significantly higher than oocyte donors (*U *= 4152.500, *P *= 0.005; Fig. 1), but both groups provided rather low scores with 75% of scores <7 for sperm donors and <5 for oocyte donors (scale range 0–16).

### Openness about having donated oocytes/sperm

**Figure 2.**
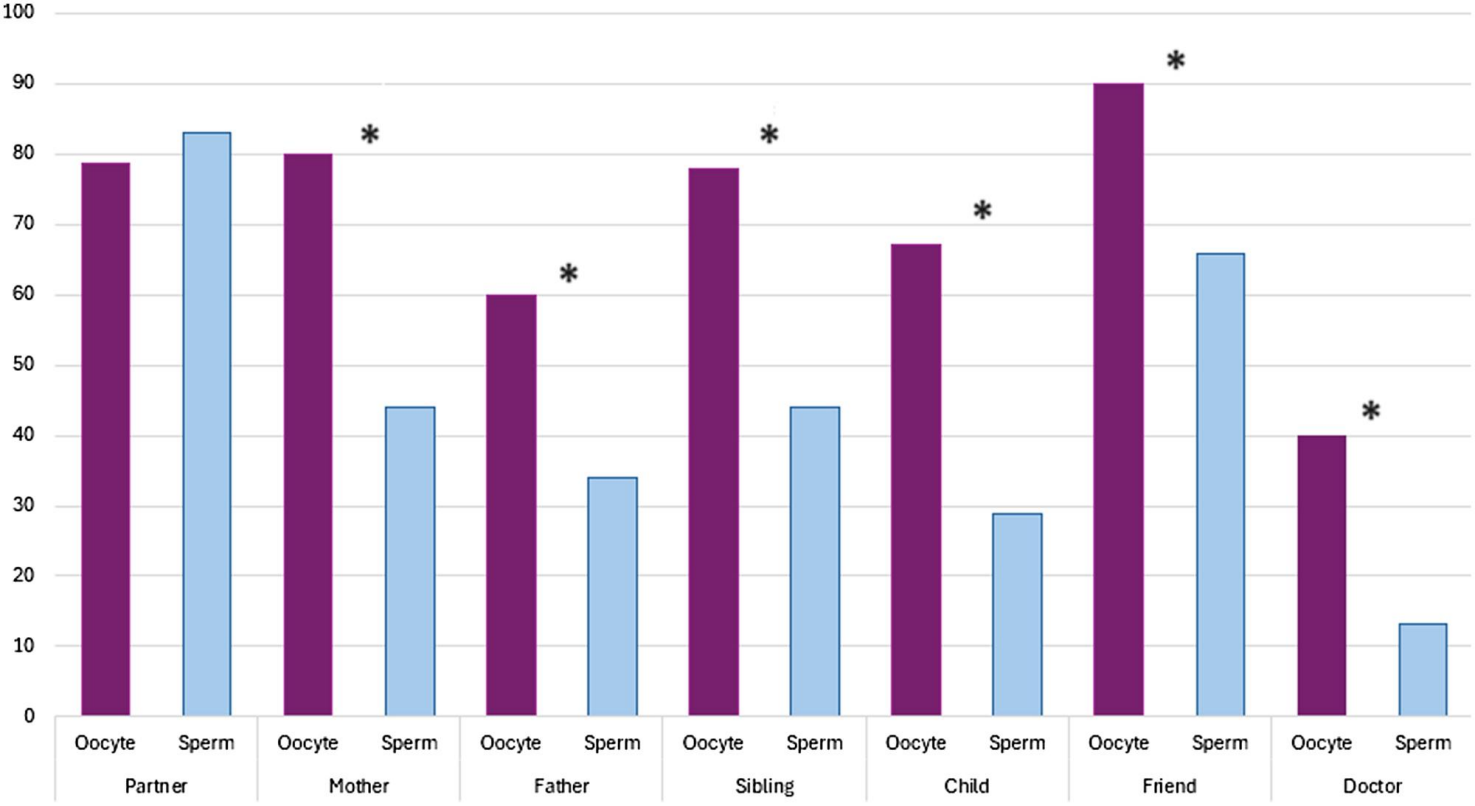
**Openness among oocyte and sperm donors 14–17 years post-donation.** Bars represent the percentage of donors reporting having told the listed persons about the donation. For the variables Partner and Child, only donors having indicated having a partner/child were included. *denotes statistical significance at the 0.001 level.

A majority of both oocyte and sperm donors had talked to other people about having donated. While the percentage of donors who had told their partner did not differ between the groups (*P* = 0.524), for all other potential confidants disclosure rates were higher among oocyte than sperm donors (*P*’s < 0.05 for all analyses; Fig. 2).

## Discussion

The present study found that with the potential release of their identity approaching, most Swedish open-identity donors were positive towards future contact with DCO. More than half wished for professional support regarding how to handle such contact and a vast majority wanted to be notified of the release of their identity.

At the time of donation, the prospect of future identity-release may seem rather abstract. For the donors in the current study, potential request for their identity was only a few years away. At the present SSGD follow-up 14–17 years post-donation, a vast majority of donors indicated that they would like to receive clinic notifications regarding the release of their identity. This is a marked increase compared to findings at the previous SSGD follow-up 5–8 years post-donation (Isaksson *et al.*, 2014) when roughly half of donors wanted such notifications. These changes may indicate that the need for notification increases as the prospect of identity-release becomes more real. It is also possible that donor attitudes have been influenced by the increased media coverage of gamete donation during the past few years (Bolt *et al.*, 2021). There was little agreement among donors as to whether notification should be conducted in advance or after identity-release. Receiving information in advance may have the advantage of allowing more time for the donor to prepare him/herself and family members for potential contact attempts by DCO.

The majority of both oocyte and sperm donors were positive towards being contacted by DCO, which is in line with previous findings from open-identity donors (Daniels *et al.*, 2005; Ekerhovd *et al.*, 2008; Graham *et al.*, 2016; Scheib *et al.*, 2017; Miettinen *et al.*, 2019). As studies have shown that DCPs value the opportunity to make contact with donors and to learn about their genetic background (Jadva *et al.*, 2010a; Scheib *et al.*, 2017; Lampic *et al.*, 2022; Widbom *et al.*, 2024), the present results are encouraging. Analysis of participants’ free-text responses showed the donors’ emphasis on considering and adapting to the needs of the DCO in relation to future contact. This is in line with previous reports of a tendency of donors to downplay their own needs and rights in favour of those of DCPs (Isaksson *et al.*, 2014; Miettinen *et al.*, 2019; Bolt *et al.*, 2021; Nordqvist and Gilman, 2022) and recipient families (Goedeke *et al.*, 2023). Most donors were either positive or neutral towards DCPs meeting members of their own family, and comments by some donors indicated that they intend to take the wishes of their family members into account and are mindful of how their potential contact with DCO may affect those close to them. Some of the comments alluded to a perceived risk of complications, which aligns with previous reports of jealousy or fear of harm to donors’ own family (Jadva *et al.*, 2010b; Daniels *et al.*, 2012; Kirkman *et al.*, 2014).

While counselling during the donation process has been found to play a crucial role for donors’ understanding of the life-long implications of donating (Hammarberg *et al.*, 2008; Visser *et al.*, 2016), donors’ need for post-donation support has not been extensively studied. More than half of the donors in the current study indicated a need for either personal counselling or written information regarding contact with offspring. This is in line with results of an interview study with open-identity sperm donors who considered it important for counselling to be available in case they would be contacted by individuals conceived from their donations (Visser *et al.*, 2016).

Overall, our results regarding the importance of a genetic parent–child link suggest that gamete donors emphasize ‘nurture’ over ‘nature’. However, sperm donors rated the genetic parent–child link as more important than oocyte donors, which is in line with similar gender differences found in the general Swedish population (Skoog Svanberg *et al.*, 2003) and among gamete recipients in Sweden (Isaksson *et al.*, 2011). It also aligns with the finding by Almeling (2011) that sperm donors often consider themselves as fathers to DCO, whereas oocyte donors emphasize that they are *not* mothers. Almeling suggests that gendered norms and practices contribute to these different views, where oocyte donors downplay the importance of genetics and instead focus on giving the recipient women the chance to become mothers. Scores on the genetic importance index were not significantly related to donors’ attitudes towards contact with DCO. However, it should be noted that the items concerned perceptions of genetics in relation to *parenthood* and not in relation to being a donor which may hamper conclusions. As previously pointed out (e.g. Mohr, 2015; Gilman, 2020), the relation between donors and the individuals conceived from their donations is not easily defined, and both parties have to explore and negotiate previously unscripted relationships in order to make sense of their connectedness.

Most donors had discussed the donation with their current partner, which is in line with previous results (Daniels *et al.*, 2012; Pennings *et al.*, 2021; Nordqvist and Gilman, 2022). However, in general, oocyte donors were more open about having donated than sperm donors. This may be related to differences in cultural perceptions of the donation types. Sperm donation is often considered a more sensitive subject and sometimes associated with sex or stereotypes of young men carelessly donating for extra money (Burr, 2009; Gilman, 2018). About half of the donors had not (yet) told their legal children about the potential existence of DCO, i.e. their genetic half-siblings. While recipient parents are typically advised to talk to their children about the donation early (e.g. Kirkman-Brown *et al.*, 2022), Nordqvist and Gilman (2022) found that fertility counsellors were less adamant in terms of their advice on disclosure to donors’ legal children. The same study also reported large variation in terms of at what age donors’ children were told about their parent having donated. Given the increased availability of direct-to-consumer DNA-tests and the fact that many donors in the current study had adult children, postponing disclosure may entail the risk of accidental discovery (Harper *et al.*, 2016; Crawshaw, 2018). In addition, not informing their legal children entails a risk of romantic involvement between individuals unaware of their status as genetically related, which is a commonly mentioned concern of DCPs (Jadva *et al.*, 2010a; Hertz and Nelson, 2018).

The results of the current study have some notable implications regarding the organization of post-donation matters within Swedish health care. First, donors should be notified about the release of their identity to individuals conceived from their donations, which has recently been included in updated practice guidelines (Stenfelt *et al.*, 2023). According to recent results regarding Swedish DCPs requesting donor information, the clinics did not routinely contact the donors regarding the release of their identity (Lampic *et al.*, 2022) nor provide DCPs with information about the donor’s stance towards potential contact (Lampic *et al.*, 2022; Widbom *et al.*, 2024). Secondly, our findings that more than half of donors expressed a need for support regarding potential future contact with DCPs highlight the need to provide adequate resources. While access to counselling both pre- and post-donation is recommended (Kirkman-Brown *et al.*, 2022; Human Fertilisation and Embryology Authority Code of Practice, 9th edition), Swedish clinics today only provide pre-donation counselling as per routine. The current results suggest that donors may benefit from written information and advice regarding contact with DCO, and from availability of counselling to manage the needs and preferences of all involved parties, including own family members. The fact that many donors had not told their legal children about having donated suggests a particular need for counselling to address this subject. Finally, in Sweden, post-donation matters are handled by the individual clinics, and there is a lack of public informational resources such as the Human Fertilization and Embryology Authority website in the UK. The results of the current study and previous studies about experiences of identity-release in Sweden (Widbom *et al.*, 2021, 2024; Lampic *et al.*, 2022) suggest a need for more centralized information. For example, a national competence centre could administer information and support to donors, DCPs, and recipients as well as to provide resources for clinics. Such services would not just benefit individuals affected by identity-release donation, but also anonymous donors and DCPs conceived before the legislative changes in 1985, for whom contact following direct-to-consumer DNA-tests has become increasingly common.

The current study is based on a national sample where all women and men accepted as gamete donors between 2005 and 2008 were approached for participation, and very high response rates were achieved at inclusion as well as at follow-up 14–17 years post-donation. These methodological strengths suggest that the sample is representative of open-identity donors in Sweden and other countries practicing altruistic donation. Potentially, the findings may to some extent pertain to commercial open-identity donors as well. The study’s longitudinal design also represents a notable strength as it provided us with the opportunity to assess donor attitudes and needs at a time point when identity-release may very soon take place. Only half of the donors used the opportunity to provide free-text responses regarding contact with DCPs and comments were often rather brief, which constitutes a limitation. Our attempts to identify factors associated with donors’ attitudes towards contact and need for support were unsuccessful, which suggests that an interview format may be needed to explore donors’ thoughts and feelings regarding potential contact with DCO.

Worldwide, increasing numbers of DCPs are reaching the age when they can obtain donor-identifying information. Open-identity oocyte and sperm donors appear to be relatively prepared for the release of their identity and potential contact attempts by individuals conceived from their donations. Our findings highlight that contemporary gamete donation has the potential for life-long consequences not only for recipients and DCPs but also for donors and their families. Therefore, it is imperative that donors have access to adequate informational resources and post-donation counselling.

## Data Availability

The data underlying this article will be shared on reasonable request to the corresponding author.
